# Researcher Perspectives on Publication and Peer Review of Data

**DOI:** 10.1371/journal.pone.0117619

**Published:** 2015-02-23

**Authors:** John Ernest Kratz, Carly Strasser

**Affiliations:** California Digital Library, University of California Office of the President, Oakland, CA, United States of America; University of Illinois at Chicago College of Applied Health Sciences, UNITED STATES OF AMERICA

## Abstract

Data “publication” seeks to appropriate the prestige of authorship in the peer-reviewed literature to reward researchers who create useful and well-documented datasets. The scholarly communication community has embraced data publication as an incentive to document and share data. But, numerous new and ongoing experiments in implementation have not yet resolved what a data publication should be, when data should be peer-reviewed, or how data peer review should work. While researchers have been surveyed extensively regarding data management and sharing, their perceptions and expectations of data publication are largely unknown. To bring this important yet neglected perspective into the conversation, we surveyed ∼ 250 researchers across the sciences and social sciences– asking what expectations“data publication” raises and what features would be useful to evaluate the trustworthiness, evaluate the impact, and enhance the prestige of a data publication. We found that researcher expectations of data publication center on availability, generally through an open database or repository. Few respondents expected published data to be peer-reviewed, but peer-reviewed data enjoyed much greater trust and prestige. The importance of adequate metadata was acknowledged, in that almost all respondents expected data peer review to include evaluation of the data’s documentation. Formal citation in the reference list was affirmed by most respondents as the proper way to credit dataset creators. Citation count was viewed as the most useful measure of impact, but download count was seen as nearly as valuable. These results offer practical guidance for data publishers seeking to meet researcher expectations and enhance the value of published data.

## Introduction

### Data sharing

In 1985– almost 30 years ago– Stephen Ceci surveyed 847 scientists and concluded “it is clear that scientists in all fields endorse the principle of data sharing as a desirable norm of science” [1]. This endorsement has not weakened over the decades; more than 65% of faculty at California Polytechnic State University (Cal Poly) affirmed the importance of data sharing in 2010 [2], as did 94% of the researchers in the United Kingdom (UK) surveyed by the Expert Advisor Group on Data Access (EAGDA) in 2013 [3]. The respondents in 1985 survey endorsed data sharing “to allow replication and extension of one’s own findings” [1], and enabling replication and (re)use is still the principal motive behind data sharing. The reproducibility problem plaguing science in the scholarly [4–6] and mainstream [7–9] press could be addressed, in part, by opening underlying data to scrutiny [10, 11]. Beyond confirming previous analyses, reuse of existing data cuts research costs [12] and allows new questions to be addressed [13, 14]. Despite the apparent enthusiasm for data sharing in principle, Ceci alleges that in practice, “something is amiss in the academy.”

Researchers frequently fail to make data available, even when they support the idea or are obliged to do so. Alsheikh-Ali et al. examined 351 articles and found that 59% did not satisfy the data availability requirements of the journal that published them [15]. Vines et al. requested data from 516 articles published between 1991 and 2011 and obtained it less than half (47%) of the time [16]. Researchers themselves agree that this is a problem. In 1985, 59% of scientists surveyed by Ceci complained that their colleagues were disinclined to share data [1]. Twenty-five years later, 67% of respondents to an international survey by the Data Observation Network for Earth (DataONE) affirmed that “[l]ack of access to data generated by other researchers or institutions is a major impediment to progress in science” and 50% felt that their own research had suffered [17]. That same year, fewer than half of the 65% of Cal Poly faculty who agreed that data sharing is important followed through to share their own data [2].

Why do researchers who believe in the importance of sharing data fail to carry through? Previous surveys unearthed a number of reasons: concern about the ethical and legal issues around human subject data, mistrust that others have the expertise to use the data appropriately, hope of wringing additional articles from the data, and fear that the data will be “stolen” without credit or acknowledgment. For example, researchers brought up ethical concerns in reports from the Research Information Network (RIN) in 2008 and EAGDA in 2014; in the 2014 report, this was the second most frequently mentioned constraint (by 55% of respondents) [3, 18]. The risk of losing publications from premature sharing came up in 60% of a series of interviews of United States (US) scientists in 2012, more than any other risk; fear of “data theft” was mentioned in 32% of the interviews [19]. However, by far the most consistent reason given is that preparing and documenting data to a high enough standard to be useful just takes too much time.

In the UK, the RIN report described lack of time as a major constraint [18], and it was mentioned by 66% of respondents to EAGDA, more than any other constraint [3]. Time was brought up by 44% of respondents to Kim and Stanton’s survey [19], more than any other cost. It was the most frequent reason for not sharing data in the multidisciplinary DataONE survey (named by 54% of respondents) [17] and the second most frequent in a follow-up survey of astrobiologists (named by 22%) [20]. Time investment was the second most frequently raised objection to data sharing in a 2012 survey of biodiversity researchers and the “most violently discussed obstacle” in associated interviews [21].

Although the process of preparing and documenting data for sharing could undoubtedly be streamlined with better planning, education, and tools, it will always take time and effort. The underlying problem is that this time and effort is not rewarded. Lack of acknowledgment was the third most popular objection in the biodiveristy survey and ∼ 2/3 of respondents would be more likely to share if they were recognized or credited when their data is used. In the EAGDA report, 55% of respondents said that lack of tangible recognition and rewards constrains data sharing, and at least 75% of respondents felt that the UK Research Excellence Framework (REF) does not recognize data to some or great extent relative to publications, but that it should. The need to compensate researchers who share data with scholarly prestige is a major driver of the movement toward data publication.

### Data publication

Data publication appropriates familiar terminology (“publication,” “peer review”) from the scholarly literature in order to insinuate data into the existing academic reward system [22–24]. The model of data publication that most closely mimics the existing literature is the data paper. Data papers describe datasets, including the rationale and collections methods, without offering any analysis or conclusions [25, 26]. Data papers appear in existing journals like *F1000Research* and *Internet Archaeology* as well as new dedicated journals such as *Earth System Science Data*, *Geoscience Data Journal* [27], and Nature Publishing Group’s *Scientific Data*– which describes itself concisely as “a publication venue that credits scientists who share and explain their data” [28]. Data papers are invariably peer-reviewed based on the dataset; its description; and whether the two form a complete, consistent, and useable package [23]. The appeal of data papers is straightforward: they are unquestionably peer-reviewed papers, so academia knows how (if perhaps not how much) to value them.

However, other data-publishing approaches abound. Data publishers include repositories such as Dryad (http://www.datadryad.org/), figshare (http://figshare.com/), and Zenodo (http://zenodo.org/) where researchers can self-deposit any kind of research data with light documentation requirements and minimal validation. Dryad requires that data be associated with a “reputable” publication, while figshare and Zenodo are completely open. Domain-specific repositories frequently have more stringent documentation requirements and access to the domain knowledge needed for thorough evaluation. For instance, the National Snow and Ice Data Center (NSIDC) evaluates incoming data in a complex process involving both internal reviewers with technical expertise and external peers with domain knowledge [29]. As a final example, Open Context publishes carefully processed and richly annotated archaeology data, some of which passes through editorial and peer review [30]. One thing that all of these publishers have in common is that they endeavor to make datasets formally citable (in part through assignment of stable identifiers) as a means to credit the creators.

The variety of forms of data publication attests to a general shortage of consensus on what, exactly, it means to publish data. Noting a lack of both consensus and interest on the part of researchers, the RIN report of 2008 adopted a deliberately minimal definition: “making datasets publicly available” [18]. While eminently practical, this definition does not do much to distinguish publication from sharing (except for ruling out certain channels) or to advance its prestige. More recently, Callaghan et al. (2012) suggested distinguishing between published (available), and Published (also citable and peer-reviewed) data [31]. We have argued that the consensus in the scholarly communications community– publishers, librarians, curators– is that published data is openly available, documented, and citable, but that what kind of validation (if any) is required to qualify is still an open question [32]. It is easy to forget, however, that what data publication means to the scholarly communication community is substantially irrelevant. The point of calling data made public through whatever particular process “published” is to exploit the meaning of the word to researchers; the important definition is what data publication means to them. This paper surveys researchers to explore these kinds of semantic gaps between the scholarly communication community and researchers and seeks to ensure that researcher expectations are more obvious, so that data publishers can maximize the return on their efforts.

Researchers have been surveyed about sharing data many times this decade, but not about data publication [2, 3, 17, 19, 33–36] The RIN report of 2008 is the most recent survey to ask researchers about data publication; while the conclusions are undeniably valuable it uses the term broadly enough that it is difficult to make any distinction between attitudes towards sharing data and publishing it [18]. Consequently, open questions abound: What would a researcher expect data publication to mean? What about peer review of a dataset? Do current models satisfy those expectations? What potential features of a data publication would be useful for evaluating the quality of the data? For evaluating the contribution of the creator(s)? To get this critical perspective we conducted an online survey of active researcher perceptions of data publication.

## Results

### Demographics

We collected responses to an online survey of data publication practices and perceptions in January and February of 2014 and received 281 unique responses. Because we distributed the survey solicitation via social media and email lists and did not contact most recipients directly, we cannot estimate with any accuracy how many researchers received the solicitation or calculate a response rate. Our analysis was restricted to the 249 (81%) respondents who we deemed to be active researchers (described in Table 1). Researchers from 20 countries responded, but most were affiliated with institutions in the US (79%, *n* = 197). The institutions were largely academic (85%, *n* = 204); 94% (*n* = 191) of those were focused on research rather than teaching. By discipline, the largest response was from biologists (37%), followed by archæologists (13%), social scientists (13%), and environmental scientists (11%). We heard from researchers across the academic career spectrum: 41% (*n* = 102) were principal investigators/lab heads, 24% (*n* = 61) postdocs, and 16% (*n* = 41) grad students. We saw few significant differences in responses between disciplines or roles, so we have presented the results in aggregate. For significance testing, we consolidated subdisciplines into 8 high-level disciplines. Given the number of respondents, this survey should have 80% power to detect small effects by chi square (*χ* ^2^) test (size Φ_*C*_ = 0.17) and 95% power to detect medium-small effects (Φ_*C*_ = 0.22) [37]. For breakdown of data by discipline or role, see the full– except for redactions to preserve anonymity– raw dataset published in the University of California’s Merritt repository [38].

**Table 1:** Demographics.

		Percent	Count
Discipline	Biology	37	91
	Archaeology	13	31
	Social science	13	32
	Environmental science	11	27
	Physical sciences	7	18
	Earth science	5	13
	Computer science	4	11
	Mathematics	1	3
	Other	9	21
Role	Principal investigator	41	102
	Postdoc	24	61
	Graduate student	16	41
	Technician	11	28
	Other	7	17
Highest degree	Doctorate	76	186
	Masters	17	41
	Bachelors	8	19
Institution	Academic: research-focused	76	191
	Government	6	14
	Academic: teaching-focused	5	13
	Nonprofit	5	12
	Academic: medical school	4	9
	Commercial	2	4
	Other	2	6

Demographic breakdown of the 249 researchers whose responses are analyzed here.

### Background knowledge

**Fig 1:**
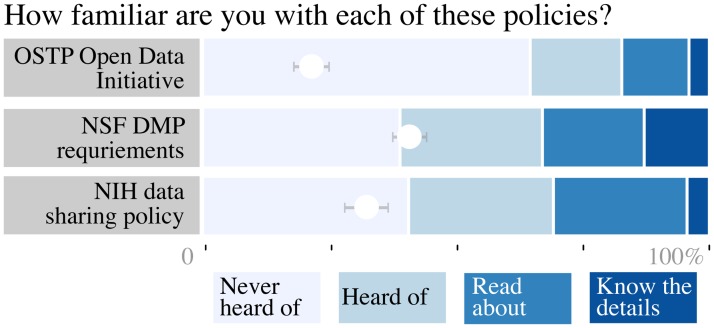
Researchers are generally unfamiliar with data-related funder policies. Respondents based at US institutions self-reported their familiarity with three government funder policies: the Whitehouse OSTP Open Data Initiative (*n* = 197), NSF Data Management Plan requirements (*n* = 197), and the NIH data sharing policy (only biologists included, *n* = 76). White dots show the mean familiarity for each item; error bars depict bootstrapped 95% confidence intervals.

We asked a number of questions to assess engagement and familiarity with data sharing and publication (Fig. 1). Respondents rated their familiarity with three US federal government policies related to data sharing and availability. Because these policies are specific to the US, we restricted this part of our analysis to respondents who work there. Respondents were most familiar with the National Science Foundation (NSF)’s Data Management Plan requirement [39]. Fewer than than half had heard of the United States Office of Science and Technology Policy (OSTP) Open Data Initiative [40]. Although the directive will eventually affect virtually all researchers who receive US government funding, awareness is most likely low because concrete policies have not been implemented yet. The much older National Institutes of Health (NIH) data sharing policy [41] was enacted 11 years ago, but only four biologists (5%) claimed to know all the details, fewer than the 18 (24%) who had never heard of it.

The recent rapid proliferation of data journals led us to ask about them specifically. A free text box was provided for respondents to list any data journals that they could name. Only 40 respondents (16%) named any data journals. *Ecological Archives* was the most frequently named, by 16 respondents. The second most frequent response was Nature Publishing Group’s *Scientific Data* (named by 14), even though it had not started publishing at the time of the survey. *Earth System Science Data (ESSD)* (*n* = 7), *Biodiversity Data Journal* (*n* = 6) and *Geoscience Data Journal* (*n* = 5) followed. A number of respondents listed non-journal data publishers: figshare (*n* = 6), Dryad (*n* = 3), and Zenodo(*n* = 1).

### Data sharing mechanisms

Data publication is a relatively new and unfamiliar concept to researchers, but most do have experience with and opinions about data sharing, and we explored those briefly before moving on to publication. Many respondents (56%, *n* = 140) said that it is very important to share the data that underlies a study; differences between disciplines were not statistically meaningful (*χ* ^2^ = 39.1, *p* = 0.18). Most have experience sharing their data (68%, *n* = 168) or reusing another researcher’s shared data (61%, *n* = 151). Of the researchers who shared, 58% (*n* = 98) saw their data reused by someone and 62% (*n* = 61) of those reuse instances led to a published paper. Most of the respondents who reused data published a paper with it (69%, *n* = 104).

**Fig 2:**
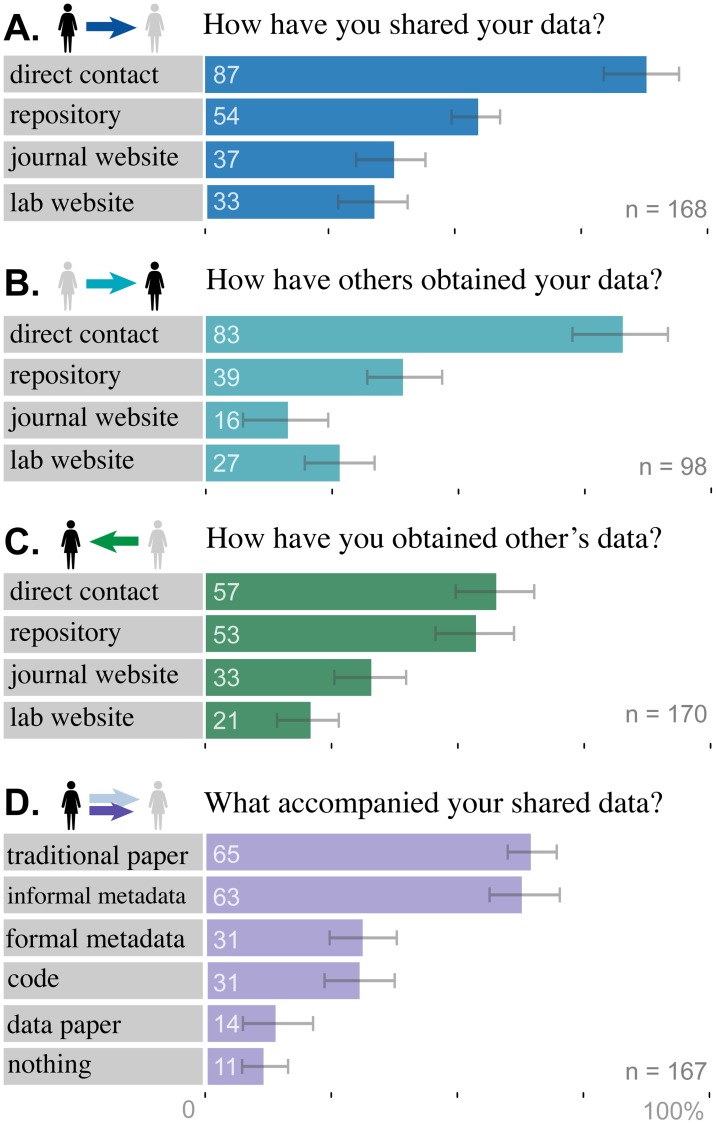
Researchers primarily share data in response to direct contact (e.g. via email). Respondents who shared data indicated (A.) the channels they used to share their data, (B.) the channels others used to obtain the data, and (D.) how they documented the data. (C.) Respondents who used others data indicated the channels through which they obtained the data. Error bars depict bootstrapped 95% confidence intervals.

Because some, but not all, means of sharing data satisfy the availability requirements of data publication [32], we asked researchers with data sharing experience about the mode of transmission (Fig. 2A–C). The supplied answer choices were the four methods for external data sharing that emerged in interviews by Kim and Stanton (2012): email/direct contact, personal website, journal website, and database or repository [19]. Email/direct contact was the most frequently reported method for sharing: 87% (*n* = 146) of the respondents who shared data did so directly, 82% (*n* = 82) were aware of other researchers obtaining their data directly, and 57% (*n* = 86) of the respondents who reused data obtained it directly. The predominance of direct contact is probably in part an artifact of awareness– respondents necessarily know when they give someone their data directly, but they may not be notified when someone downloads it from a repository or website. Eight respondents (5%) wrote-in that they had obtained data through a channel we had not considered: extracting data from the text, tables, or figures of a published paper.

### Credit for sharing data

**Fig 3:**
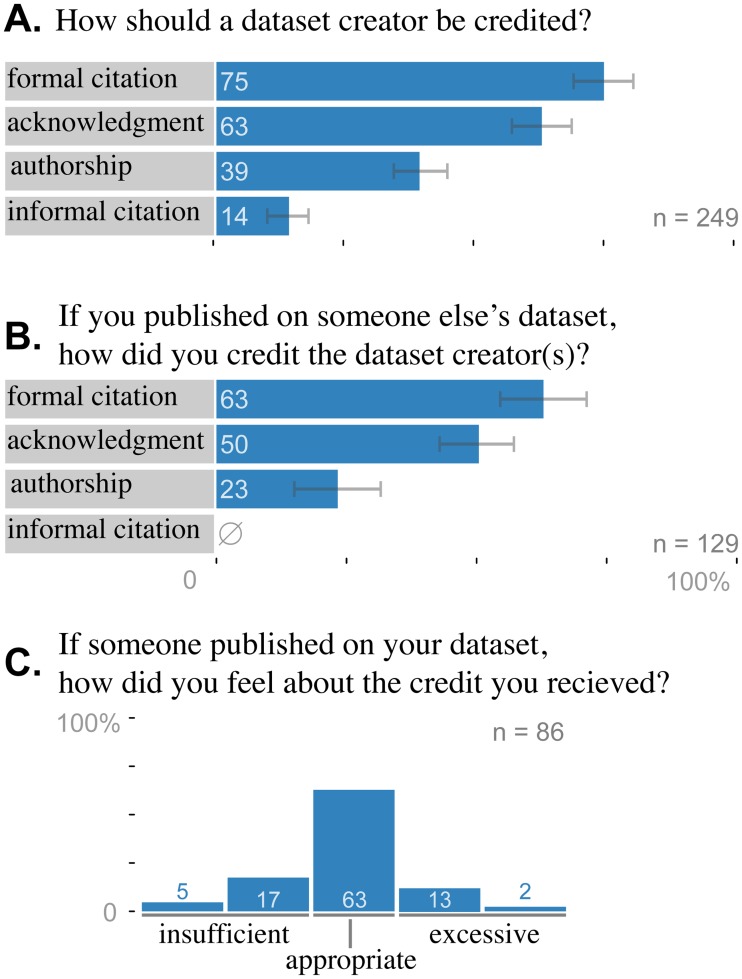
Formal citation is the preferred method of crediting dataset creators. Respondents indicated (A.) how a dataset creator should be credited, (B.) how they actually credited a dataset creator in the past, and (C.) how satisfied they were with the credit they received the last time someone else published using their data. (A., B.) Respondents could select more than one item for each question. Error bars depict bootstrapped 95% confidence intervals.

Rewarding data creators is a primary goal of data publication, so we asked how a dataset creator should be credited by a reuser (Fig. 3A.). The most common answer, from 83% (*n* = 126) of respondents, was formal citation in the reference list. Acknowledgment also ranked highly at 62% (93). Most (30 out of 34) respondents who gave a free-text answer wrote some variant on “it depends,” often citing one of two factors: the publication status of the dataset (e.g. “depends on whether the data is already published”) and the role of the data in the paper (e.g. “authorship if data is [the] primary source of analysis, otherwise acknowledgment”). Because previous studies reported differences in citation practices between disciplines [17, 18, 33], we tested whether different disciplines responded differently (omitting Mathematics because the *n* was too low to reliably test). Following the approach of Tenopir et al. (2011), we performed separate *χ* ^2^ tests for each of the four provided answer choices. Using a significance cutoff corrected for multiple hypothesis testing of *α* = 0.05/4 = 0.0125, we did not detect a difference between disciplines (*χ* ^2^ ≤ 16.4, *p* ≥ 0.022).

We also asked respondents who had published with shared data how they actually credited the creator. Reported practice fit well with theory: formal citation was the most popular method (63%, *n* = 81), followed by acknowledgment (50%, *n* = 70). A notable distinction was that while a few respondents (16%, *n* = 24) said that it was appropriate to cite data informally in the body of the text, none admitted to actually doing it (Fig. 3B.).

Many researchers fear that shared data might be used by “data vultures” who contribute little and don’t acknowledge the source [19]. To assess how realistic this fear is, we asked respondents whose data had been reused for a publication whether they felt adequately credited. Most (63%, *n* = 54) felt satisfied, and a combined 78% (*n* = 67) felt the credit was appropriate or excessive. This left 22% (*n* = 13) who were unsatisfied; only 2 (2%) felt that the credit was “very insufficient.” These differences in satisfaction could derive from different attitudes toward appropriate credit. To test this, we collapsed responses into three categories (insufficient, appropriate, and excessive) and tested for independence with each of the four provided answers in data sharing credit, but none of the relationships were statistically meaningful (corrected *α* = 0.0125, *χ* ^2^ ≤ 3.26, *p* ≥ 0.20).

### Expected features of data publication and peer review

**Fig 4:**
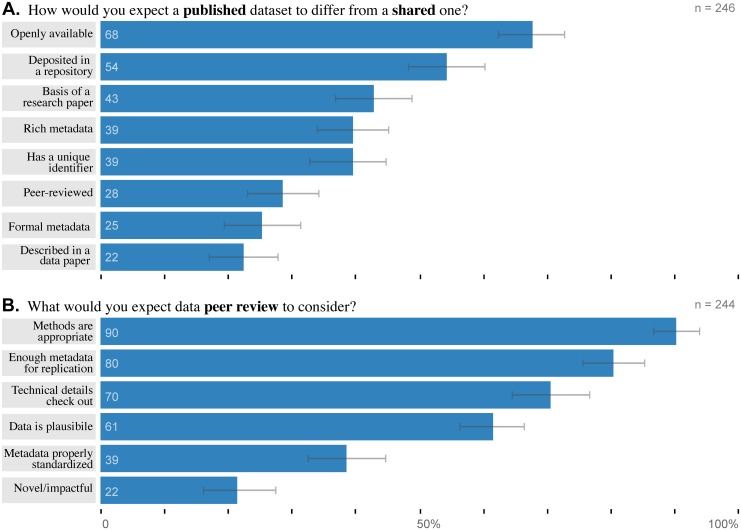
Researcher expectations of data publication center on availability, not peer review. Respondents conveyed the expectations raised by the terms (A.) publication and (B.) peer review in the context of data. Respondents could select more than one item for each question. Error bars depict bootstrapped 95% confidence intervals.

The central question we hoped to answer is what “data publication” and “data peer review” actually mean to researchers. We decomposed the prevalent models of data publication into a set of potential features and asked respondents to select all the features that would distinguish a “published” dataset from a “shared” one (Fig. 4A). The most prevalent expectations relate to access: 68% (*n* = 166) expect a published dataset to be openly available and 54% (*n* = 133) expect it to be in a repository or database. Substantially more researchers expected a published dataset to be accompanied by a traditional publication (43%, *n* = 105) than by a data paper (22%, *n* = 55). Only a minority of 29% (*n* = 70) expected published data to have been peer-reviewed.

Much of the prestige of scholarly publication derives from surviving the peer review process. It is natural, then, that many data publication initiatives model their validation process on peer review and employ the term for its prestige and familiarity. However, it is not obvious exactly how literature peer review processes and criteria should be adapted for data or what guarantees it should make. We asked what researchers expect from data peer review, providing a selection of considerations that data reviewers might take into account (Fig. 4B). The most common responses sidestepped examination of the data itself; 90% (*n* = 220) of respondents expected evaluation of the methods and 80% (*n* = 196) of the documentation. There was little (22%, *n* = 53) expectation that data reviewers would consider novelty or potential impact.

We tested for differences in expectations of both data publication and peer review among disciplines and between research roles. No significant differences between roles emerged. The only two significant differences among disciplines related to structured metadata: discipline had a significant effect on expectation of formal metadata in the publication process (corrected *α* = 0.006, *χ* ^2^ = 33.0, *p* = 2.6 × 10^−5^) and consideration of standardized metadata in peer review (corrected *α* = 0.008, *χ* ^2^ = 26.7, *p* = 3.8 × 10^−4^). In both cases, the most notable distinctions were the expectations of a large fraction of environmental scientists: 63% (compared to 25% in the population as a whole) for publication and 73% (compared to 39%) for peer review. This popularity among environmental scientists may be driven by the use of a mature metadata standard in the field, Ecological Metadata Language (EML) [42].

**Fig 5:**
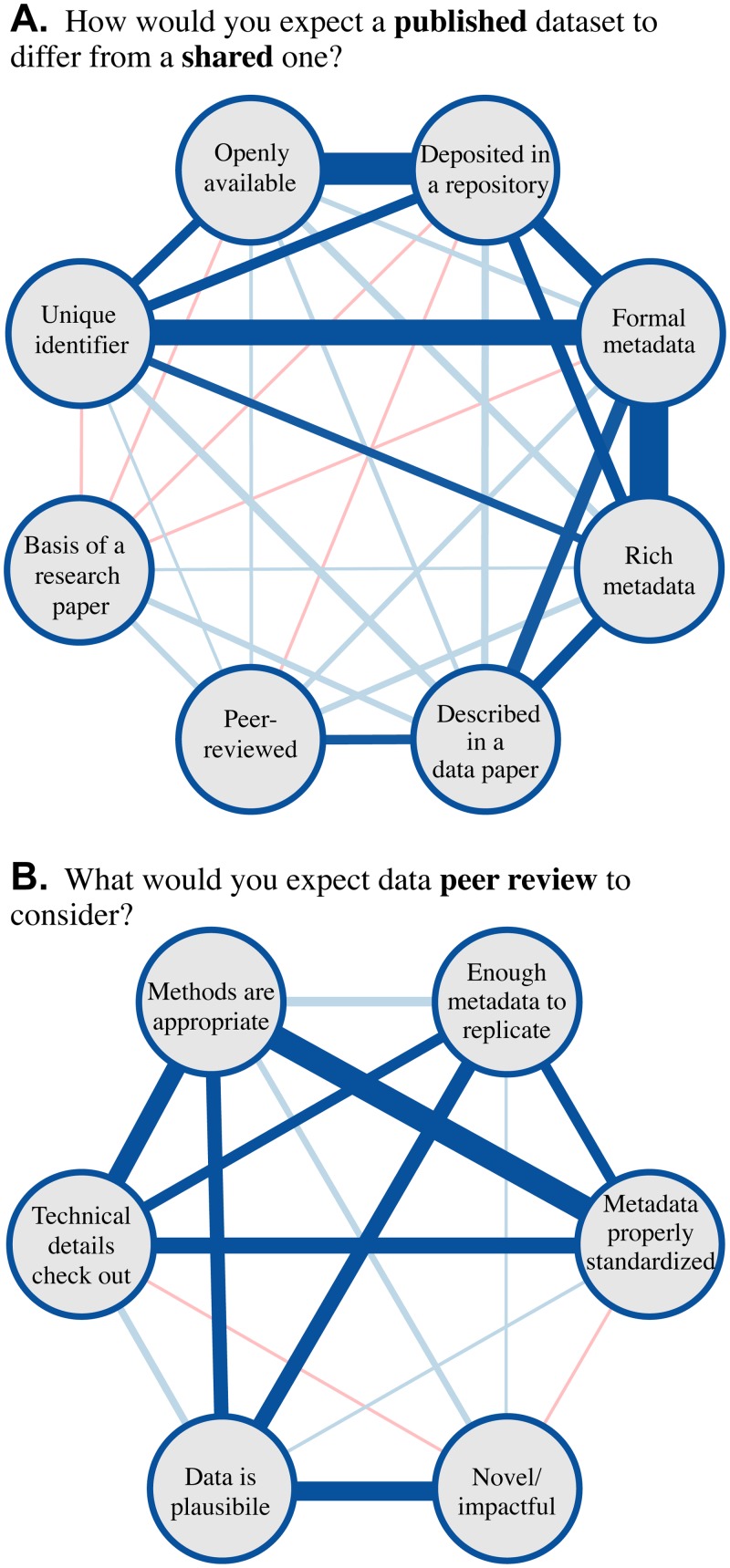
Researchers have coherent expectations of data publication and peer review. Graph of relationships between the researcher expectations shown in Fig. 4. Nodes are potential (A.) publication features or (B.) peer review assessment. Edges depict relationship strength as measured by odds ratio. Blue edges show positive relationships, red are negative. Dark edges are significant at the *α* = 0.05 level by Fisher’s exact test, with correction for multiple hypothesis testing to (A.) *α* = 0.0018 and (B.) *α* = 0.0033.

To learn whether respondents selected data publication features or peer-review assessments independently or as coherent constellations of ideas, we performed Fisher exact tests of independence between every pair of features (Fig. 5). Within data publication, we found a dense set of statistically significant associations among items related to access and preservation (at the *α* = 0.05 level, corrected to *α* = 0.0018). For example, repository deposit was linked to openly availability (*OddsRatio* = 9.55, *p* = 7.6 × 10^−14^), assignment of unique identifier, (*OR* = 3.33, *p* = 1.33 × 10^−5^), and both formal (*OR* = 4.49, *p* = 3.94 × 10^−6^) and rich (*OR* = 3.83, *p* = 1.14 × 10^−6^) metadata. Formal and rich metadata were themselves linked (*OR* = 12.5, *p* = 2.7 × 10^−14^). Formal metadata was also linked to assignment of a unique identifier, which is sensible in that an identifier is meaningless without metadata (*OR* = 7.92, *p* = 5.05 × 10^−11^). Another carrier for metadata, a data paper, was linked to both rich (*OR* = 3.64, *p* = 4.22 × 10^−5^) and formal (*OR* = 4.30, *p* = 1.32 × 10^−5^) metadata. Data papers were the only item associated with peer review (*OR* = 3.00, *p* = 0.0011). Traditional papers had no significant associations at all; the closest was with data paper (*OR* = 1.93, *p* = 0.023).

Potential considerations during peer review are also linked significantly (with a corrected cutoff of *α* = 0.0033). Three assessments were strongly interlinked: from appropriate methods to standardized metadata (*OR* = 7.91, *p* = 0.00081) to technical evaluation (*OR* = 4.56, *p* = 3.4 × 10^−6^) and back (*OR* = 5.9, *p* = 7.9 × 10^−5^). Plausibility correlated with other factors that require domain expertise: appropriate methods (*OR* = 4.51, *p* = 0.0014), adequate documentation (*OR* = 4.87, *p* = 2.5^−6^), and novelty/impact (*OR* = 5.50, *p* = 1.1 × 10^−5^). Plausibility was the only association for novelty/impact.

### Valued data-publication features

**Fig 6:**
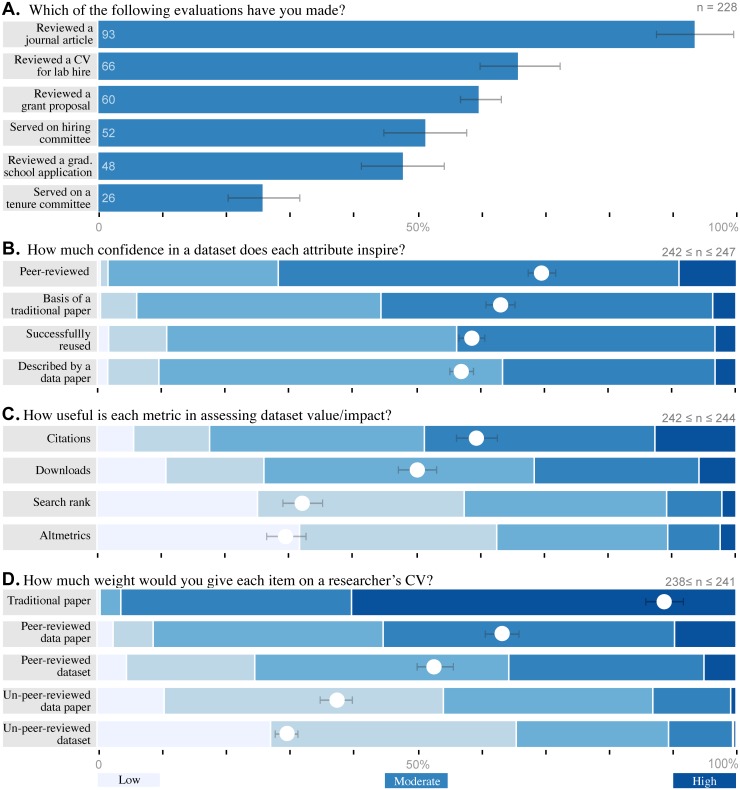
Researchers trust and value peer review highly. (A.) Respondents reported their past experience evaluating other researchers in each context; respondents could select more than one item. Respondents reported (B.) how much trust each data publication feature inspires, (C.) how useful each metric would be for assessing impact, and (D.) how valuable a CV item each kind of data publication would be. White dots show the mean response for each item; error bars depict bootstrapped 95% confidence intervals.

Validation of published data facilitates use only if potential users trust the means of assessment. To learn which means researchers trust, we presented respondents with four possible features and asked how much to rate how much confidence each would confer (Fig. 6B). All four inspired at least some confidence in most researchers (ranging from 89% to 98%). Respondents trusted peer review above all else: 72% (*n* = 175) said it conferred high or complete confidence and only 2% (*n* = 4) would feel little or no confidence. The second most trusted indicator was knowledge that a traditional paper had been published with the data; 56% (*n* = 137) would have high or complete confidence. Reuse of the data by a third party came in third, with 43% (*n* = 106). Description by a data paper was the least convincing at 37% (*n* = 89) high or complete confidence, although reuse inspired little or no confidence in more respondents (11%, *n* = 25).

Beyond reuse, data publication should reward researchers who create useful datasets with credit. To that end, we asked what metrics researchers would most respect when evaluating a dataset’s impact (Fig. 6C). Respondents considered number of citations to be the most useful metric; 49% (*n* = 119) found citation count highly or extremely useful. Unexpectedly, a substantial 32% (*n* = 77) felt the same way about number of downloads. The distinction between citation and download counts shrinks to 9% if the comparison is made at the level of at least somewhat useful (82% versus 73%). Only a minority of respondents considered search rank (42%, *n* = 102) or altmetrics (37%, *n* = 91) to be even somewhat useful.

Even before quality or impact enter consideration, the prestige associated with publishing a dataset is influenced by its format. We distilled a multiplicity of data publications formats to four generic models- with or without a data paper and with or without peer review– and asked respondents how much each would contribute to a researcher’s curriculum vitæ (Fig. 6D). As a point of comparison, respondents also rated the value of a traditional paper; 60% (*n* = 145) give one a great deal of weight and another 36% (*n* = 87) give it significant weight. The most valuable data publication model was data published with a peer-reviewed data paper, but even that was only given a great deal of weight by 10% (*n* = 23), although another 46% (*n* = 109) gave it significant weight. A peer-reviewed dataset with no paper dropped to 5% (*n* = 12) giving a great deal of weight, while an un-peer-reviewed data paper dropped to 1% (*n* = 2). Thus, peer review outweighed having a data paper as a factor. A substantial, 27% (*n* = 65) would award an un-peer-reviewed dataset no weight at all. For this question, which explicitly addressed evaluation of dataset creators, we were particularly interested in the 26% (59) of survey respondents who had experience on a tenure and promotions committee. We compared their responses to each feature with those who had not served on a committee by *χ* ^2^, but found no significant relationships (corrected *α* = 0.01, *χ* ^2^ ≤ 8.39, *p* ≥ 0.078).

## Discussion

### Demographics, statistical power, and bias

Although this survey was international in scope, most of the respondents were affiliated with institutions in the United States. The respondents here (84% North American) resemble those of the DataONE survey [17] (73% North American); many of the previous surveys were conducted entirely in the US [1, 2, 19]. The in-depth reports prepared by EAGDA [3] and the RIN [18] were carried out in the UK, where a single assessment framework, the REF dominates, creating a significantly different environment in terms of credit. The bulk of our responses (85%) came from academic institutions, which is similar to DataONEs 81% [17]. Ceci’s initial survey was academic [1], and Scaramozzino’s was conducted entirely at a single teaching-oriented university [2]. In this respect, the population here is quite comparable to previous surveys.

Researchers in all of the major roles in academia and a variety of disciplines responded. In terms of role, our respondents again resemble those of the DataONE survey. There, 47% were professors and 13.5% grad students; here, 41% were principal investigators and 16% grad students [17]. Most other surveys were restricted to principal investigators. An exception, the EAGDA survey, still mostly (69%) heard from principal investigators [3]. Our largest response was from biologists (37%), followed by archaeologists (13%), social scientists (13%), and environmental scientists (11%). DataONE heard mostly from researchers in its area of focus, environmental sciences and ecology (36%), followed by social science (16%) and biology (14%) [17]. Scaramozzino’s survey included a high proportion of physicists and mathematicians, but 18% of respondents were biologists [2]. The EAGDA survey was heaviest in biomedical fields, such as epidemiology and (26.8%), genetics/genomics (20%), but also featured 31.4% social scientists [3].

Whereas DataONE uncovered statistically distinct data sharing attitudes between respondents in different disciplines, we did not. The effect sizes observed in tables 21 and 22 of Tenopir et. al (2011) [17]– which most closely parallel the questions about appropriate credit for sharing data presented here– range from an effect size of Φ_*C*_ = 0.11 to 0.17. This survey should have 80% sensitivity to an effect size at the top of this range, Φ_*C*_ = 0.17, so we find it plausible that the detection of statistically meaningful distinctions in one survey and not the other could an artifact of the difference in statistical power (from an *n* of 1329 vs. 249) rather than a reflection of real differences in the respondent populations. However, 0.17 is comfortably a “small” effect, so we are unlikely here to have missed large or even moderate effects by chance [37].

As is the case for many of the previous surveys, participation was voluntary and open, so our sample may be biased toward researchers with an interest in data sharing and publication. However, a high proportion of respondents (84%) did not name any data journals, especially relative to the 40% of EAGDA respondents who were unfamiliar with the format [3]. That and the low awareness of US federal policies (e.g., 35% of US respondents had never heard of the NSF data management plan requirement and 62% had never heard of the OSTP Open Data Initiative) suggest that our respondents are not atypically invested in these issues.

### Data publication

The RIN report of 2008 concluded that “…‘publishing’ datasets means different things to different researchers” [18] and we found that little has changed. Even the most frequently named defining feature in this survey, open availability, was only chosen by ∼ 2/3 of respondents. One respondent simply wrote “terms are confusing.” However, the emergence of systematic relationships between some of the features demonstrates that the responses were not utterly confused. We observed two, arguably three, independent concepts of data publication.

The most widely held concept centers on present and future access. Open availability tightly correlates with the second most frequent feature, repository deposit. Repository deposit correlates with three other conceptually related features (unique identification, rich metadata, and formal metadata), and numerous interconnections unite all five of these features. This concept of publication maps well onto virtually all present data publication implementations, including lightweight approaches like figshare and Zenodo.

The second concept lingers from the pre-digital days of scholarly communication: published data is data that has been used or described in a traditional journal article. Nearly half (43%) of the respondents chose “basis of a research paper” as a defining feature of data publication. Surprisingly, this and the previous concept did not compete, but were instead almost completely independent. The traditional paper concept reflects how researchers speak (e.g. to “publish an experiment” is to publish a research paper that uses the experiment), but does not match the conversation in the scholarly communication community, where data that had been used or described but not made available would not be considered to have been published and, conversely, data that has been made available but never used in a research paper might be. This mismatch is a potential source of misunderstanding that the scholarly communication community should be aware of.

The third concept, not entirely independent from the first, is that a published dataset is one that has been described by a data paper. Data papers correlate with peer review and both kinds of metadata, but not with features related to the disposition of the data (e.g. open availability or repository deposit), even though virtually all data paper publishers require repository deposit. Data papers conferred less trust than any other feature, but only by a small margin: 36% of respondents derive high or complete confidence from a data paper, compared to 44% from successful reuse. Respondents regarded data papers as much less valuable than traditional research papers: 60% would give traditional paper a great deal of weight, but only 10% would value a data paper that highly. Only 16% had been able to name a data journal at the start of the survey, and data papers may come to be valued more as awareness spreads; one respondent wrote “I’ve never heard of this, but it sounds fantastic.” Alternatively, research communities may conclude that data papers *should* be valued less. Already, 55% of respondents gave a data paper significant (or higher) value, and that may ultimately be appropriate. Data papers clearly add perceived value to a dataset, but not as much as peer review.

### Validating published data

Quality control via peer review is integral to traditional scholarly publication so it is no surprise that, in reference to data publication, the RIN noted “[t]here is, for some, also an implication that the information has been through a quality control process” [18]. Even in regard to novel material like data, researchers trust the traditional scholarly publication process: our respondents trusted peer review and use in a research paper more than any other indicators of quality. However, less than half expected published data to have been used in a published research paper and only one third expected it to have been peer reviewed. We conclude, with the RIN, that researchers don’t have a clear idea what quality control to expect from published data. In this uncertainty, the research and scholarly communication communities are in perfect agreement. How, and how extensively, to assess data quality is the least settled of the many open questions surrounding data publication, and different initiatives take a variety of approaches, including collecting user feedback, distinct technical and scientific review, and closely modeling literature peer review [32].

Peer review establishes the trustworthiness of dataset and elevates its perceived value more than any other factor in this survey. Despite one respondent’s remark that “I have never heard this term applied to a dataset and I don’t know what it means,” expectations of peer review were more consistent than of publication. Whereas only 68% of respondents selected even the most popular feature in the question on data publication, 90% agreed that they expect data peer review to include evaluation of collection and processing methods. In fact, half of the peer review assessments were selected by more than 68% of respondents.

Unsurprisingly, a majority of respondents expect assessments that require domain expertise, i.e. that peer review involve review by peers in their field. Assessment of plausibility was linked with three other assessment that require domain expertise: method evaluation, adequacy of metadata for replication, and potential novelty/impact. The high (80%) expectation that peer review of data includes peer review of its documentation/metadata suggests that researchers are aware of the critical importance of documentation for data reuse and replication. That and the low (22%) expectation that peer review consider novelty/impact are in line with current data journal peer review processes and guidelines [32]. However, our survey question focused on the aspects of a data publication that might be assessed, not the review process, and peer review expectations might be satisfied through any number of pre- or post-publication processes. We conclude that models of data publication without peer review are unlikely to confuse researchers, but that peer review greatly enhances both reuse and reward. Furthermore, assessment processes that at least meet the expectations of peer review will be critical for data publications to attain a status at all comparable to that of journal articles.

The idea that “data use in its own right provides a form of review” [43] is frequently expressed in the conversation around data publication. Reuse could be documented through citations from research papers to the dataset or direct feedback from researchers who used the data. Based on past experiences, we were surprised that successful reuse did not inspire more trust; both peer review and “basis of a traditional paper” inspired slightly more confidence than reuse. It is worth nothing that serving as the basis of a research paper by the dataset creator is itself evidence of successful use, just not by a third party. However, respondents did consider citations to be the most useful metric for assessing value/impact. This apparent contradiction could result from evaluating trustworthiness and impact differently or from different concepts of “successful” reuse and reuse that that results in a citation. The combined value of enhancing trust and establishing impact makes tracking dataset citations eminently worthwhile, but still no substitute for peer review.

### Credit for publishing data

The scholarly communication community agrees that data should be cited formally in the reference list [44], but this is rarely actually done [45–47]. In a 1995 survey of 198 papers that used published social science datasets, 19% cited the dataset with at least the title in the reference list [45]. A followup 17 years later found that only 17% of papers meeting even this low standard, showing that practice has not improved [47]. The most common actual approach is informal citation in the methods or results section of the paper; 30.8% of papers in 1995 and 69.2% in 2012 included the dataset title somewhere in the text. Notwithstanding this dismal state of practice, researchers agree that the correct approach is formal citation; 95% of respondents to DataONE said that formal citation was a fair condition for data sharing, 87% of astrobiologists said the same, and 71% of biodiversity researchers said they would like their data to be cited “in the references like normal publications” [17, 20, 21]. Here too, formal citation was the most popular response to both how a dataset creator should be credited and how the respondent actually credited data creators. No respondents admitted to citing data informally in the text. This apparent disconnect between what is observed in the social science literature and self-reported practice could arise in any of a number or ways: it may be that social science is not a representative discipline, that occasions when respondents cited data formally are easier to bring to mind, or that researchers define dataset reuse differently than the authors of the literature surveys. For instance, a biologist who uses a sequence from GenBank and mentions the accession number in the methods section of the paper might not think of that activity as data reuse warranting a formal citation. Beyond notions of credit, formal data citations are useful to the 71% of respondents to the EAGDA survey who already track use of their datasets “through details of publications generated using the data” [3]. We conclude that researchers are aware of the benefits of formal data citation and suggest that data citation efforts focus on implementation rather than persuasion.

While respondents deemed citation the most useful metric of dataset value, they also attached high value to download counts. These preferences align with the practices reported in the EAGDA survey, where 43% of respondents tracked downloads of their datasets [3]. In the present scholarly communication infrastructure, repositories can count downloads much more easily than citations; citations are preferable, but downloads are the “low hanging fruit” of data metrics. In comparison to download counts, appreciation of altmetrics (e.g. mentions in social media or the popular press) was low: only one third of respondents found them even somewhat useful in assessing impact. Altmetrics for research articles are still being developed, so it is not surprising that researchers are unsure what they might signify for data. For data publishers, there is certainly no harm in providing altmetrics– and a majority of respondents did find them at least slightly useful– but they are unlikely to have much impact in the short term.

Researchers see the time required as the biggest cost to data sharing, but the risk they most fear is that “data vultures” will strip the data for publications without adequately acknowledging the creator(s) [19]. To learn how well-founded these fears are, we asked respondents how satisfied they were with their credit the last time someone published using their data. The majority felt that the credit was appropriate, but the fraction that felt shortchanged (22%) is too large to ignore, and we must conclude that this dissatisfaction is a real problem. Whether the problem is ultimately with the way dataset creators are credited or the way dataset creators *expect* to be credited is for research communities to decide. We can say that there was no significant difference in how satisfied and dissatisfied respondents thought dataset creators should be credited, so the variability in satisfaction was most likely driven by variability in credit received rather than the respondent’s expectation. As data publication takes shape, the problem can be reduced by solidification of community norms around data use, increased prestige for dataset creators, and better adoption of formal data citation.

### Practical conclusions

The results of this survey offer some practical guidance for data publishers seeking to meet researcher expectations and enhance the value of datasets. Above all else, researchers expect published data to be accessible, generally through a database or repository; this fits well with current practice and, indeed, with the idea of publication at its most fundamental. The research and scholarly communication communities agree that formal citation is the way to credit a dataset creator, and a number of steps can be taken to encourage this practice. Data publishers should enable formal citation (e.g. by assigning persistent identifiers and specifying a preferred citation format), and article publishers should encourage authors to cite data formally in the reference list. Data publishers should track and aggregate citations to their datasets to the extent feasible; at a minimum, they should publicize download counts, which are less valued by researchers but easier to implement. Data papers enhance dataset value, but much of the value of a peer-reviewed data paper can be obtained by peer review alone. Peer review is not integral to data publication for researchers, but it remains the gold standard of both trustworthiness and prestige. Repositories and databases can make data more useful to both creators and users by incorporating peer review, whether by managing the process themselves or integrating with peer-reviewed data journals. While many aspects of data peer review are unresolved, two clear expectations that should be met are that true peers will supply domain expertise and that evaluation of metadata will play a significant role.

## Methods

### Ethics statement

All results were drawn from a survey approved by the University of California, Berkeley Committee for Protection of Human Subjects/Office for the Protection of Human Subjects (protocol ID 2013-11-5841). Respondents completed the survey anonymously. Researchers affiliated with the University of California (UC) could supply an email address for follow-up assistance with data publication, but neither the fact of affiliation nor any UC-specific information was used in this analysis.

### Survey design and distribution

The survey contained 34 questions in three categories: demographics, data sharing interest and experience, and data publication perceptions (S1 Text). Demographic questions collected information on respondent’s country, type of institution, research role, and discipline. Questions to assess respondent’s existing knowledge of data sharing and publication focused on knowledge of several relevant US governmental policies and an invitation to name data journals. Data publication perceptions consisted of “mark all that apply” questions concerning definitions of data publication and peer review and Likert scale questions about the value of various possible features of a data publication. The number of required questions was kept to a minimum. Some questions were displayed dynamically based on previous answers. Consequently, *n* varies considerably from question to question.

The survey was administered as a Google Form, officially open from January 22 to February 28 of 2014; two late responses received in March were included in the analysis. Solicitations were distributed via social media (Twitter, Facebook, Google+), emails to listservs, and a blog post on Data Pub [32].

### Data processing and analysis

Although the topic of the survey is benign and identification would be unlikely to negatively impact respondents, light anonymization was performed prior to analysis and release of the response data. UC affiliation and answers to UC-specific questions were redacted. Respondent locations were grouped into United States and “other.” Questions that related to US policies were analyzed based on US respondents only; one questions about the NIH was analyzed based only on US biologists Fig. 1. Sub-disciplines with fewer than three respondents were re-coded with the corresponding discipline. Listed data journal names were standardized manually, and free text answers to other questions were replaced with “other.” Because few questions were required, “mark all that apply” questions with no reply at all were considered to be skipped.

After anonymization, responses were filtered for analysis. Because the goal of the survey was to learn about researchers as distinct from the scholarly communication community, we exempted from analysis anyone who self-identified as a librarian or information scientist. To restrict the analysis to active researchers only, anyone who affirmed that they had not generated any data in the last five years was exempted; the 90 who respondents did not answer this question were retained. Finally, respondents without at least a Bachelors Degree were filtered out. In total, 32 respondents were removed before analysis, some by multiple criteria.

Statistical significance was tested using Fisher’s exact test where possible (i.e., for 2x2 tables) and contingency *χ* ^2^ in all other cases [48]. A statistical significance cutoff of *α* = 0.05 was used. When testing for e.g., effects of discipline or prior experience, each answer choice was tested separately, then the Bonferroni correction for multiple hypothesis testing was applied to adjust *α* for that question; this is a conservative approach that may not detect subtle differences. Mathematicians were omitted from *χ* ^2^ significance testing for effects of discipline because their low *n* led to unacceptably small expected count numbers. Odds Ratios (ORs) [49] and Fisher’s exact test p-values were used to assess the relationships between items depicted in Fig. 5; to enable meaningful comparison, all ORs were calculated in the direction that yielded a value ≥ 1.

The robustness of all significant results was confirmed using a jackknife procedure in which the test was repeated systematically with each respondent removed; in no cases did the absence of a single respondent raise the p-value above the designated significance threshold. Error bars represent 95% basic bootstrap confidence intervals (10,000 resamples) of the mean response in Fig. 5 and the percentage of positive responses in all other figures. To facilitate comparison, *χ* ^2^ effects were measured as Cramér’s V (Φ_*C*_) [50].

Analysis was performed using IPython [51], Pandas [52], and Numpy [53]. Graphs were prepared using matplotlib [54] and formatted with Adobe Illustrator. Statistical power calculations were performed using G*Power 3.1 [55]. An IPython notebook containing the code used for data analysis is available at Zenodo [56].

## Supporting Information

S1 Text Survey Instrument. (PDF)
