# Correction: Researcher Perspectives on Publication and Peer Review of Data

**DOI:** 10.1371/journal.pone.0123377

**Published:** 2015-04-02

**Authors:** The PLOS ONE Staff

The DOI in the Data Availability statement is incorrect. The data are available from the University of California Merritt repository (http://dx.doi.org/10.5060/d8rp4v).
