# Supplementary material for: Researcher Perspectives on Publication and Peer Review of Data
Source: PLoS One. 2015 Feb 23;10(2):e0117619. doi: 10.1371/journal.pone.0117619 (PMC4338305; doi:10.1371/journal.pone.0117619)
Supplement: S1 Text — (PDF) [file pone.0117619.s001.pdf]

Data Publication Survey

* Required

Consent

You are being asked to participate in a research study in which the California Digital Library is investigating researcher attitudes around the sharing and publication of research data. Your responses will help us and other organizations develop services for data sharing that satisfy the actual wishes of researchers.

If you decide to participate, we will record your responses from the following survey. The survey should take about 20 minutes to complete. In addition to demographic information, questions relate to researcher practices and attitudes around data sharing, re-use of published data, validation of datasets, and credit for dataset authors. No sensitive items are included in our survey, and therefore we do not anticipate that your participation poses any personal or professional risk.

Your responses will be recorded anonymously, unless you choose to provide your contact information. In that case, information linking you to your responses will be restricted to only two researchers. We will maintain confidentiality when reporting survey results. Results will be compiled in a peer-reviewed publication, presentation(s), and report(s). Absolute confidentiality cannot be guaranteed, because research documents are subject to subpoena.

There is no direct benefit to you anticipated from participation in this study. However, results from this study will be used in the development of services to encourage and support sharing of research data.

Your participation in this research is voluntary, and you may decline to participate without risk. While it is useful to be complete in your responses to the survey, you are free to withdraw from the survey at any time.

If you have any questions about this research project, please contact:

John Kratz
Postdoctoral Fellow
California Digital Library
415 20th St., 4th Floor
Oakland, CA 94612
john.kratz@ucop.edu

Background Knowledge and opinions

How familiar are you with each of the policies below?

1. **NSF Data Management Plan Requirements for your discipline**
   Mark only one oval.

   - Know all the details
   - Read about it
   - Heard of it
   - Never heard of it

2. **NIH Data Sharing Policy**
   Mark only one oval.

   - Know all the details
   - Read about it
   - Heard of it
   - Never heard of it

3. **Office of Science and Technology Policy (OSTP) Open Data Initiative**
   Mark only one oval.

   - Know all the details
   - Read about it
   - Heard of it
   - Never heard of it

4. **University of California Open Access Policy**
   Mark only one oval.

   - Know all the details
   - Read about it
   - Heard of it
   - Never heard of it

Are you familiar with any data journals? If so, what titles?

A data journal is a journal that publishes datasets and/or data papers.
A data paper describes a dataset, including the collection and processing methods used and the rationale, but doesn't provide any analysis or attempt to draw any conclusions.

5.

Data Sharing

6. **How important is it to share data that underlies a published study?**
Mark only one oval.

	1	2	3	4	5	
Very Important	◯	◯	◯	◯	◯	Not at all important

7. **How should a researcher who shares data be credited?**
(e.g. by email, submitting to database, or as supplemental material) Check all that apply.
Check all that apply.

- [ ] Authorship on paper
- [ ] Acknowledgement in the paper
- [ ] Data cited in the reference list
- [ ] Data cited informally in the text of the paper
- [ ] Not credited
- [ ] Not applicable
- [ ] Other:

8. **Have you ever shared your data with anyone outside your research group and collaborators?** *
(e.g. by email, submitting to database, or as supplemental material)
Mark only one oval.

◯ Yes
◯ No *Skip to question 18.*
◯ Not sure / Not applicable *Skip to question 18.*

Data Sharing Experience

9. **Have you shared data in any of the following ways?**
Check all that apply.
Check all that apply.

- [ ] Email / direct contact
- [ ] Personal or lab website
- [ ] Journal website (as supplemental material)
- [ ] Database or repository
- [ ] Don’t know
- [ ] Not applicable
- [ ] Other: ....

10. **Which of the following did you include with your data?**
Check all that apply.
Check all that apply.

- [ ] A traditional research paper based on the data (with analysis and conclusions)
- [ ] A data paper describing the data (without analysis or conclusions)
- [ ] Informal text describing the data
- [ ] Formal metadata describing the data (e.g. as XML)
- [ ] Computer code used to process or generate the data
- [ ] Shared with no additional documentation
- [ ] Other: ....

11. **Have you been listed as an author on a peer-reviewed paper in the last 5 years?**
Mark only one oval.

- ( ) Yes
- ( ) No
- ( ) Not sure / Not applicable

12. **Have you generated any data in the last 5 years?**
Mark only one oval.

- ( ) Yes
- ( ) No
- ( ) Not sure / Not applicable

13. **If yes, roughly what percentage of the data is publicly available?**
(e.g. in a public database, as supplemental material, or on your lab's website) Include newly generated data that is not yet public, but that will be made available soon.

....

14. **Has your data been re-used by anyone outside your research group / collaborators?** *
(e.g. re-analysed, used to draw new conclusions, or incorporated into a larger dataset)
Mark only one oval.

- Yes
- No *Skip to question 18.*
- Not sure / Not applicable *Skip to question 18.*

Reuse of your data

15. **How did they get your data?**
Check all that apply.
Check all that apply.

- [ ] Email / direct contact
- [ ] Personal or lab website
- [ ] Journal website (as supplemental material)
- [ ] Database or repository
- [ ] Don’t know
- [ ] Other: ....

16. **Did they publish a paper using some of your data?**
Mark only one oval.

- Yes
- No
- Not sure / Not applicable

17. **If yes, how did you feel about the amount of credit you were given?**
(e.g. authorship, acknowledgment, citation)
Mark only one oval.

	1	2	3	4	5	
Very insufficient	◯	◯	◯	◯	◯	Very excessive

18. **Have you ever re-used data from another research group (not as part of an existing collaboration)?** *
(e.g. re-analysed, used to draw new conclusions, or incorporated into a larger dataset)
Mark only one oval.

- Yes
- No *Skip to question 22.*
- Not sure / Not applicable *Skip to question 22.*

Using others' data

19. **How did you get the data?**
Check all that apply.

- [ ] Email / direct contact
- [ ] Personal or lab website
- [ ] Journal website (as supplemental material)
- [ ] Database or repository
- [ ] Don't know
- [ ] Other: ____________________

20. **Did you publish a paper using the data in some way?**
Mark only one oval.

- ( ) Yes
- ( ) No
- ( ) Not sure / Not applicable

21. **If yes, how did you credit them?**
Check all that apply.
Check all that apply.

- [ ] Authorship on paper
- [ ] Acknowledgement in the paper
- [ ] Data cited in the reference list
- [ ] Data cited informally in the text of the paper
- [ ] Not credited
- [ ] Not applicable
- [ ] Other: ____________________

22. **If a colleague described one dataset as "published" and another as "shared", how might you expect the “published” dataset to differ from the “shared” one?**
Check all that apply.
Check all that apply.

- [ ] Openly available, without contacting the author(s)
- [ ] Deposited in a database or repository
- [ ] Assigned a unique identifier, such as a DOI
- [ ] A traditional research paper is based on the data
- [ ] A data paper (without conclusions) describes the data
- [ ] Packaged with a thorough description of the data
- [ ] Packaged with formal metadata describing the data (e.g. as XML)
- [ ] Dataset is "peer reviewed"
- [ ] I don't see any difference
- [ ] Other: ....

23. **If a colleague described a dataset as "peer reviewed", which of the following would you expect to have been part of the process?**
Check all that apply.
Check all that apply.

- [ ] Collection and processing methods were evaluated
- [ ] Descriptive text is thorough enough to use or replicate the dataset
- [ ] Necessary metadata is standardized (e.g., in XML)
- [ ] Technical details have been checked (e.g., no missing files, no missing values)
- [ ] Plausibility considered, based on area expertise
- [ ] Novelty/impact considered
- [ ] Other: ....

Assessing data

24. **If you were thinking about using someone else’s dataset in your work, how much confidence in the data would each of these things inspire?**
Mark only one oval per row.

	Complete confidence	High confidence	Some confidence	Little confidence	No confidence
Described in a traditional paper (with conclusions)	◯	◯	◯	◯	◯
Described in a data paper (description only)	◯	◯	◯	◯	◯
Peer-review of the dataset	◯	◯	◯	◯	◯
Re-used by others	◯	◯	◯	◯	◯

25. **In assessing a dataset's value/impact, how useful is each of the following metrics?**
Mark only one oval per row.

	Extremely useful	Highly useful	Somewhat useful	Slightly useful	Not at all useful
Number of citations					
Number of downloads					
Number of mentions in blog posts, Twitter, or popular media					
Rank in Google searches					

Assessing researchers

26. **Which of the following have you done?**
Check all that apply
Check all that apply.

- reviewed a journal article
- reviewed a grant proposal
- reviewed an application to graduate school
- reviewed a CV to hire someone for your lab
- served on a hiring committee
- served on a tenure & promotions committee

27. **In evaluating a CV, how much weight would you put on each of the following?**
Mark only one oval per row.

	A great deal	Significant	Some	A small amount	None
Traditional paper (with conclusions)					
Data paper (description only), peer reviewed					
Data paper (description only), not peer reviewed					
Standalone dataset, peer reviewed					
Standalone dataset, not peer reviewed					

Demographics

28. **Which best describes your employer/institution?**
Mark only one oval.

- Academic: research-focused
- Academic: teaching-focused
- Academic: medical school
- Government
- Nonprofit
- Commercial
- Other: ______________________

29. **Where is your employer/institution located?**

____

30. **Are you affiliated with the University of California?** *
Mark only one oval.

- Yes
- No *After the last question in this section, stop filling out this form.*

31. **What is the highest degree you hold?**
Mark only one oval.

- Highschool
- Associate
- Bachelor’s
- Masters
- Doctorate

32. **In what year did you receive your degree?**

____

33. **Which best describes your role?**
Mark only one oval.

- Principal Investigator / Lab Head
- Postdoc
- Graduate student
- Technician / Staff
- Librarian / Information Professional
- Other: ______________________

34. **Which best describes your discipline?**

Mark only one oval.

- Social science
- -Anthropology
- -Archaeology
- -Area studies
- -Economics
- -Political science
- -Psychology
- -Sociology
- Space science
- -Astronomy
- -Astrophysics
- Earth science
- -Environmental Science
- -Geology
- -Oceanography
- -Planetary Science
- Life science
- -Biochemistry
- -Bioinformatics
- -Biology
- -Evolutionary Biology
- -Neurobiology
- Chemistry
- Physics
- Computer science
- Mathematics
- Information science
- Other

University of California Researchers

35. **What Campus are you affiliated with?** *
Mark only one oval.

- UC Berkeley
- UC Davis
- UC Irvine
- UC Los Angeles
- UC Merced
- UC Riverside
- UC San Diego
- UC San Francisco
- UC Santa Barbara
- UC Santa Cruz
- UCOP

36. **Do you have a dataset you would be interested in publishing?**
Mark only one oval.

- Yes
- No

37. **If you would like someone to contact you about publishing your dataset, please supply an email address.**

Powered by
Google Forms
